# Intense atmospheric frontogenesis by air–sea coupling processes during the passage of Typhoon Lingling captured at Ieodo Ocean Research Station

**DOI:** 10.1038/s41598-022-19359-2

**Published:** 2022-09-15

**Authors:** Sinil Yang, Il-Ju Moon, Hyo-Jun Bae, Baek-Min Kim, Do-Seong Byun, Hwa-Young Lee

**Affiliations:** 1 Department of Environmental Atmospheric Sciences, Pukyong National University, Busan, Republic of Korea; 2 Typhoon Research Center, Jeju National University, Jeju, Republic of Korea; 3 Ocean Research Division, Korea Hydrographic and Oceanographic Agency, Busan, Republic of Korea

**Keywords:** Atmospheric science, Ocean sciences

## Abstract

The Ieodo Ocean Research Station (Ieodo ORS) is a fixed marine observation platform at the boundary of the Yellow and East China Seas. In 2019, a Category 4 Typhoon Lingling passed by the Ieodo ORS very closely. At that time, the Ieodo ORS observed Sea Surface Temperature (SST) cooling of 4.5 °C by vertical mixing and negative turbulent heat flux (i.e., the sum of sensible and latent heat fluxes) due to the SST cooling. In this study, uncoupled and coupled simulations were conducted to examine the role of air-sea interactions in changes in atmospheric frontogenesis around the typhoon passage. In the coupled simulation, SST cooling up to 6 °C occurred over the dangerous semicircle due to vertical mixing induced by wind stress. Strong wind stress also enhanced the SST gradient and, therefore, contributed to the formation of a steeper atmospheric frontal zone. Moreover, the comparison with reliable datasets supports the physical linkage between SST cooling and atmospheric frontogenesis by utilizing the meridional theta-e gradient and moisture convergence zone. Therefore, from the simulation results, we hope to improve our understanding of atmospheric frontogenesis by air–sea coupling processes in the future development of a coupled atmosphere–ocean modeling system.

## Introduction

Ocean observation fixed towers are invaluable platforms for exploring various oceanic, meteorological, and coastal phenomena^1^. In general, the tower-based platform is the preferred choice for direct measurement presently compared to other oceanic floating platforms such as buoys and ships^2^. Fixed observation is useful when investigating typhoon dynamics^3,4^, regional oceanographic studies^5,6^, and air-sea exchanges^7,8^. However, ocean observation towers are very rare. Few of the towers designed to observe air-sea interactions include the Yongxing air-sea flux tower in the South China Sea, Woods Hole Oceanographic Institution (WHOI) air-sea interaction tower in the North Atlantic, and the Ieodo Ocean Research Station (hereafter Ieodo ORS) in the Yellow Sea and the East China Sea. Among the three platforms, the Ieodo ORS is located in the open ocean, where typhoons occur frequently in the East China Sea^9^. It is also a unique location to study the vertical mixing of two water mass layers of the surface water and Yellow Sea Bottom Cold Water (YSBCW) during the typhoon passage event.

A strong surface wind stress induces significant sea surface temperature (SST) cooling through vertical mixing of upper ocean waters^10–13^. Tropical cyclone-induced SST cooling is related to tropical cyclone (TC) properties, such as intensity, translation speed, and size^14–18^. Among these properties, intense and slow TCs interact longer with the ocean over the TC period^19^. This atmospheric forcing contributes to enhanced vertical mixing in the upper ocean^20^ and easily triggers strong surface cooling^17–19,21–23^. Several studies have reported that this instantaneous surface cooling influences the weakening of the TC because of the enhancement of negative SST feedback to TC^24–28^. In contrast, Lin et al.^19^ stated that some fast TCs have the potential to intensify over shallow waters owing to the relatively weaker amplitude of surface cooling than slow TCs. Nevertheless, few studies^20,29^ have quantified the relationship between air and sea interaction and fast-moving TCs.

In 2019, Typhoon Lingling, a fast-moving TC (translation speed > 10 m/s), categorized as category 4, passed straight northward over the YSBCW, passing very close to the Ieodo ORS. Typhoon Lingling formed in the east of the Philippine Sea on September 2 and intensified into a severe cyclonic storm on September 4. The maximum wind speed reached approximately 61 m/s at 00 UTC on September 6, the strongest wind observed in the Republic of Korea since Typhoon Maemi in 2003. Owing to the interaction between the typhoon and the stratified ocean, Ieodo ORS measured a dramatic SST cooling of approximately 4.5 °C just about 6 h after the typhoon passed. In addition, turbulent heat fluxes (i.e., the sum of sensible and latent heat fluxes) were observed using an eddy covariance system in the Ieodo ORS. Although some flux observations were rejected to reduce abnormal information in extreme conditions through their quality control processes, the observations represent the impacts of air-sea interaction on typhoons over the Yellow Sea and East China Sea (YECS). Herein, energy exchanges in surface heat fluxes are important to TC intensity^30–32^. Thus, SST cooling is a key player in the energy exchange^33,34^, and the surface turbulent heat flux is a thermal mediator between the ocean and TCs^18^.

For the numerical investigation of the air-sea coupling, earlier studies used a fully coupled atmosphere–ocean model for TCs via a model coupling toolkit^27,35–37^. They mainly employed the Coupled-Ocean–Atmosphere-Wave-Sediment Transport (COAWST)^35^ modeling system. The COAWST includes Regional Ocean Modeling System (ROMS) and Weather Research and Forecast Model (WRF)^38^, even wave and sediment components. In the WRF model, there is a main traditional scheme for the surface layer parameterization of the revised MM5 Monin–Obukhov surface layer scheme^39^. Despite the importance of TC-induced changes in surface turbulent heat flux, the effect on the evolution of warm fronts (i.e., atmospheric frontogenesis) has not yet been studied based on the previous coupled simulations. Of note, a new extratropical cyclone with a warm front moved northward in the East China Sea after the landfall of Typhoon Lingling at 09–10 UTC on September 7. In the cyclone, the warm front is a boundary between air masses of different equivalent potential temperatures (hereafter theta-e). That is, atmospheric frontogenesis is a measure of how this front strengthens or weakens with time and is important for forecasting the development of precipitation.

The present study examines the understanding of the air–sea coupling process and its effect on atmospheric frontogenesis in the coupled atmosphere–ocean modeling system. To isolate the importance of air-sea coupling, we provide a quantitative comparison of the negative SST feedback to the atmospheric layer up to 200 hPa during the typhoon passage. In this study, we designed uncoupled and coupled experiments, NOCPL and CPL, to investigate the air–sea coupling effect using the WRF and ROMS model. The remainder of this paper is organized as follows. We discuss the ocean response to typhoons in the coupled model and the atmospheric response to the cold pool over the analysis domain. In particular, we highlight the impact of temperature gradient changes by cooling the low-level atmospheric layer on the atmospheric frontogenesis in the coupled simulation. A brief description of the coupled model, model configuration, and experimental design is provided in the “Methods” section.

## Results

### Comparison with results at the Ieodo ORS

**Figure 1:**
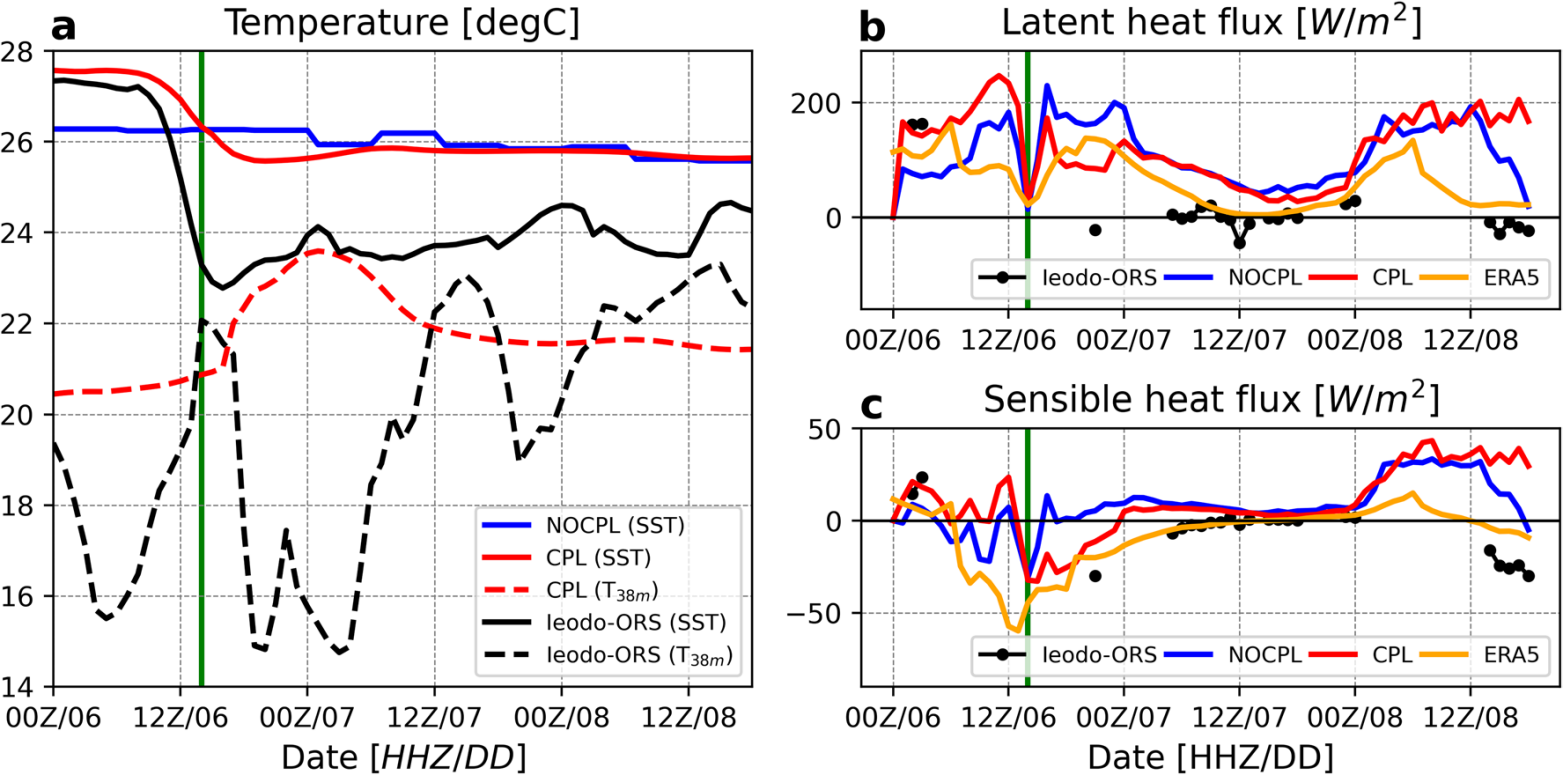
Time series of the (**a**) sea surface temperature (SST), (**b**) latent heat flux, and (**c**) sensible heat flux for observation in Ieodo ORS (black) and simulation results in NOCPL (blue) and CPL (red). The vertical thick green line denotes when the TC center was closest to the Ieodo ORS at 15Z on September 6, 2019. The T_38m_ was the bottom water temperature at a depth of 38 m. The yellow lines in the right panels were from ERA5 reanalysis.

The Ieodo ORS observed an extreme cold wake with an intense SST cooling of 4.5 °C by vertical mixing in the subsurface water column (see Fig. 1). In the CPL simulation, SST gradually decreased during the first 12 h, reached its minimum value of 25.5 °C, and then continued to maintain cold state features after 0000 UTC on September 7. As the sea surface cooled, subsurface water temperatures at 38 m depth increased until 0000 UTC on September 7. Simultaneously, the subsurface water temperature peaked at approximately 24 °C at 0000 UTC on September 7 and slowly declined. The sea-surface cooling of the coupled simulations was consistent with the observations, but the cooling was weak and slow. Not surprisingly, the NOCPL simulation did not capture any abrupt surface cooling because it used the FNL skin temperature for SST forcing.

Ieodo ORS also showed sensible and latent heat fluxes using an eddy covariance system during the typhoon period. As the SSTs continued to decrease below approximately 26 °C, the turbulent heat flux (i.e., the sum of sensible and latent heat fluxes) decreased based on the Monin–Obukhov similarity theory^40^ (see Fig. 1b, c and Eq. 2). Although the flux observations of the Ieodo ORS were massively rejected when Typhoon Lingling was closest to the Ieodo‐ORS site at 1500 UTC on September 6, the Ieodo ORS provided some evidence of reversed heat transfer from the atmosphere to the ocean when colder SST of 24 °C persisted after the typhoon passage. Here, the surface heat fluxes in the CPL simulation were mostly closer to the observations than in the NOCPL. Moreover, Figs. 1 and S3 show additional comparisons of spatial and temporal information for the surface latent and sensible heat fluxes between simulation results and ERA5 reanalysis. The ERA5 is a reliable reanalysis dataset for weather and climate investigation wherein the observations are relatively sparse. These figures confirm that the surface heat flux in the CPL simulation was reliable, which is more consistent with the ERA5 relative to the NOCPL simulation in the absence of air-sea interactions.

### Impact of the air-sea interaction on TC activity

**Figure 2:**
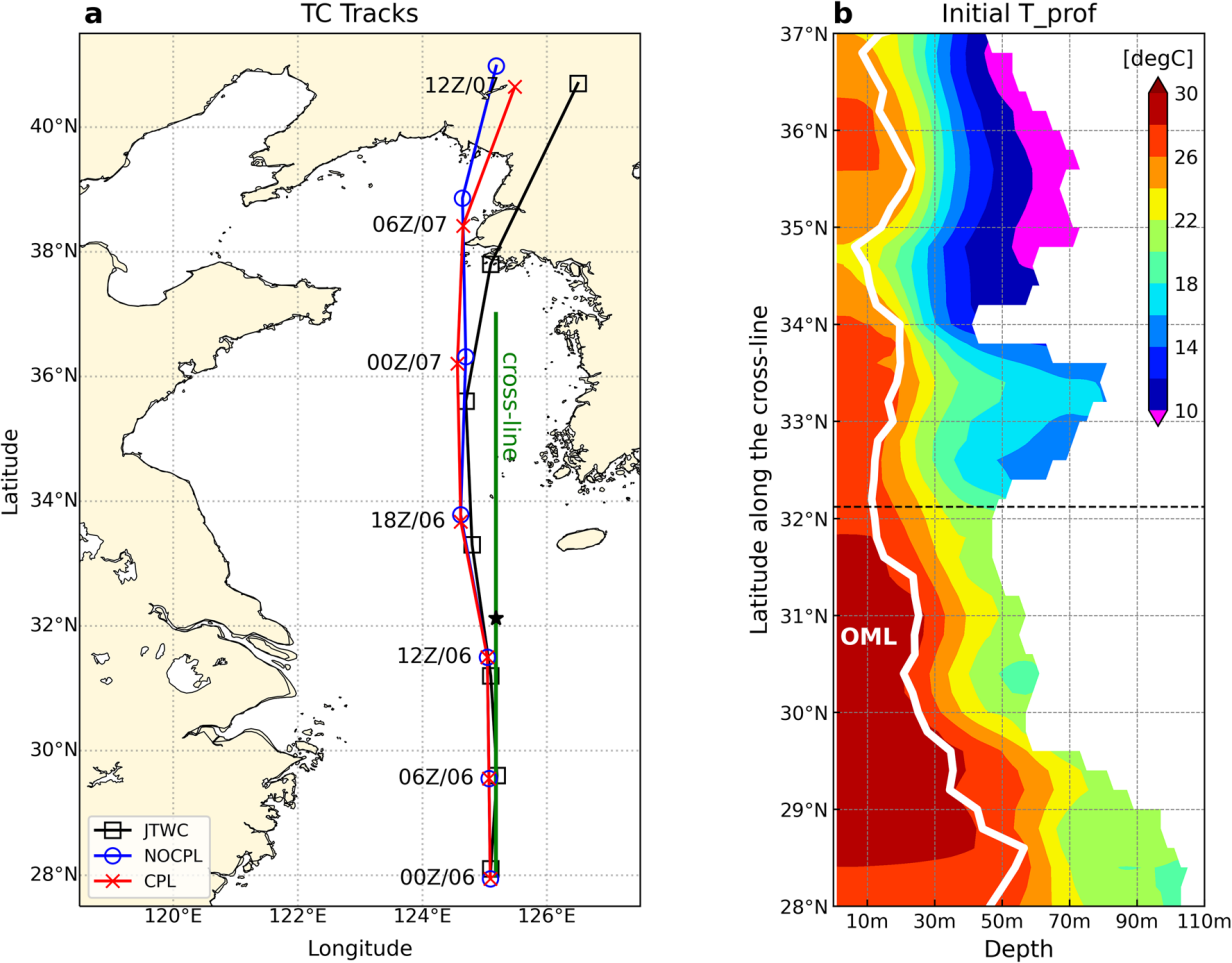
(**a**) Simulated typhoon tracks from different model configurations in the NOCPL (blue circle) and CPL (red cross) along with the JTWC reported best-track (black square) for Typhoon Lingling (2019). The meridional green line indicates the cross-line in 28–37° N at 125.18° E. The black star indicates the location of the Ieodo ORS. (**b**) The latitude-depth profile of the initial water temperature (°C; shaded) and OML depth (thick white line) at 00Z on September 6.

According to the JTWC report, Typhoon Lingling passed straight northward over the YSBCW. Two simulated TC tracks and JTWC data are shown in Fig. 2a. All simulated tracks were similar to the JTWC track before passing the YSBCW at 0000 UTC on September 7. After passing through the Yellow Sea, the two simulated tracks revealed a slight northward deviation compared to the JTWC track approaching the northern Yellow Sea and traveling parallelly, and made landfall in North Korea at the same time. There was no significant difference in the intensity between the CPL and NOCPL simulations (see Supplementary Fig. S2a, b online). The simulated typhoon was a faster-moving TC with a higher translation speed than approximately 10 m/s. The translation speed of the TC was consistently faster than the JTWC report before landfall (Fig. 2a). This result suggests that the fast translation speed in all simulations was significantly influenced by the weakening of the atmosphere–ocean interactions.

### Oceanic response in the coupled model

**Figure 3:**
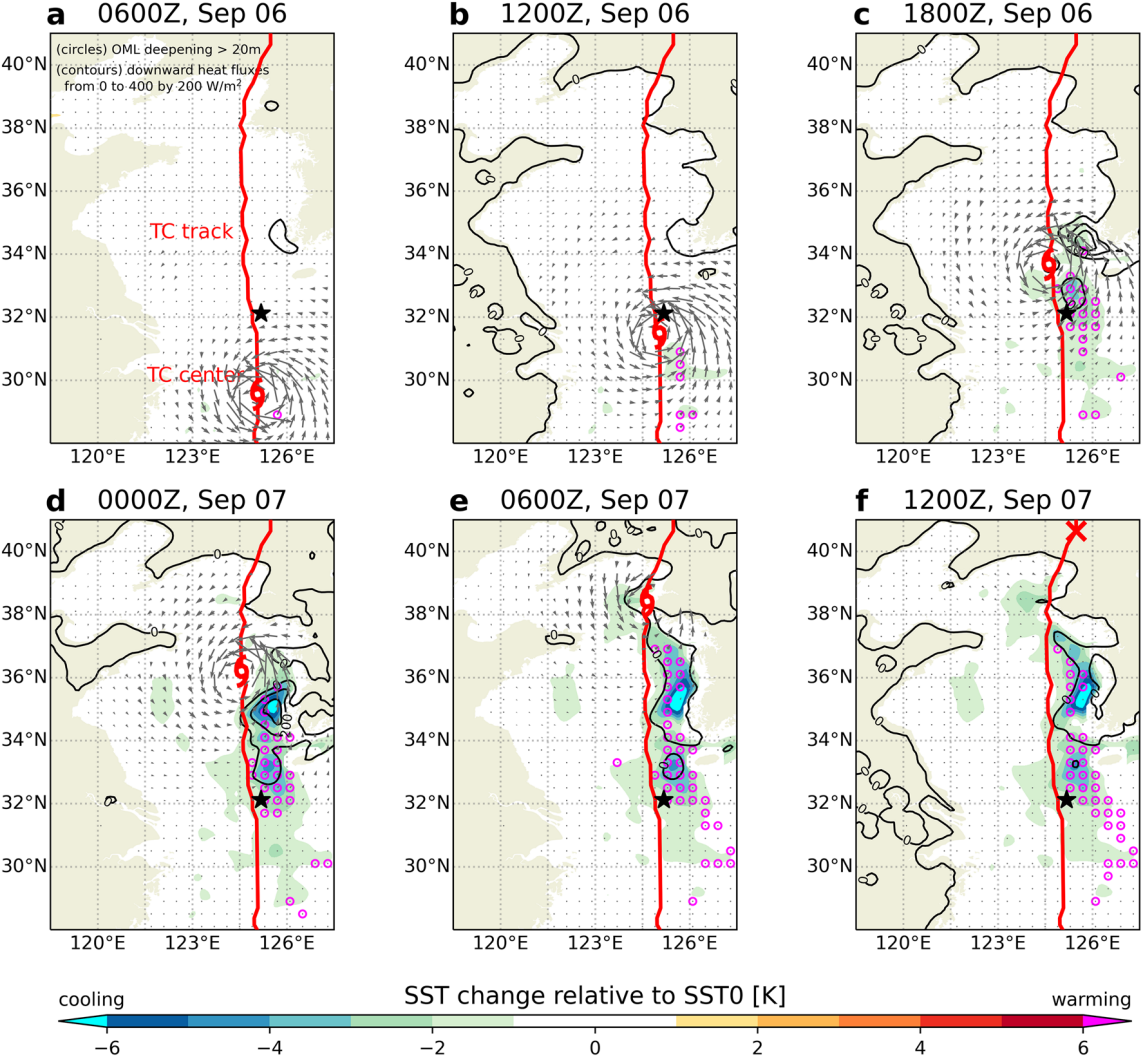
Horizontal distribution of sea surface temperature (SST) changes (shaded) relative to initial condition for the CPL simulation at 00Z on September 6 for the analysis domain every 6 h. The contours and unfilled circles denote downward (positive) turbulent heat fluxes only and OML deepening exceeding 20 m, respectively. Grey arrows denote wind stress vector (N/m^2^). Red lines and typhoon symbols indicate TC tracks and TC center location. The black star indicates the location of the Ieodo ORS.

This section focuses on understanding the oceanic response to typhoons in CPL simulation. Despite the fast translation speed, the most apparent effect along the TC passage was the remarkable SST cooling of 1–6 °C on the right side of the simulated track in Fig. 3. The TC-induced wind stress over the dangerous semicircle of the TC caused vertical mixing with a lagged response of at least 6 h in the upper ocean. For example, after the TC passed through the Ieodo ORS, the ocean mixed-layer (OML) depth increased (Fig. 3c). The OML depth, a subsurface proxy of ocean–atmosphere variability, was computed from the ocean model results using the potential density and curvature-based algorithm in Lorbacher et al.^41^. Meyers et al.^42^ reported that entrainment mixing typically accounts for most of the observed cooling of the SSTs and OML deepening due to divergent near-surface currents caused by wind stress. Most of the OML deepening occurs in the dangerous semicircle region behind the TC center, as shown in Fig. 3. The horizontal pattern of the SST changes was also consistent with that of the OML deepening. Over the YSBCW on September 7, a substantial deepening of OML that reached the closest depths to the bottom occurred (see Fig. 3d–f). In Fig. 2b, we show meridional section at 28–34° N latitude versus water depths at a 125.18° E longitude of the Ieodo ORS to identify the vertical structure of the YSBCW. The YSBCW with a minimum water temperature of less than 10 °C corresponds to 34° N to 37° N in the latitudes. This region plays an important role in SST cooling through entrainment into the upper ocean via vertical mixing. Thus, the TC-induced wind stress affects variations in the OML deepening by increasing the positive current shear along the TC passage.

**Figure 4:**
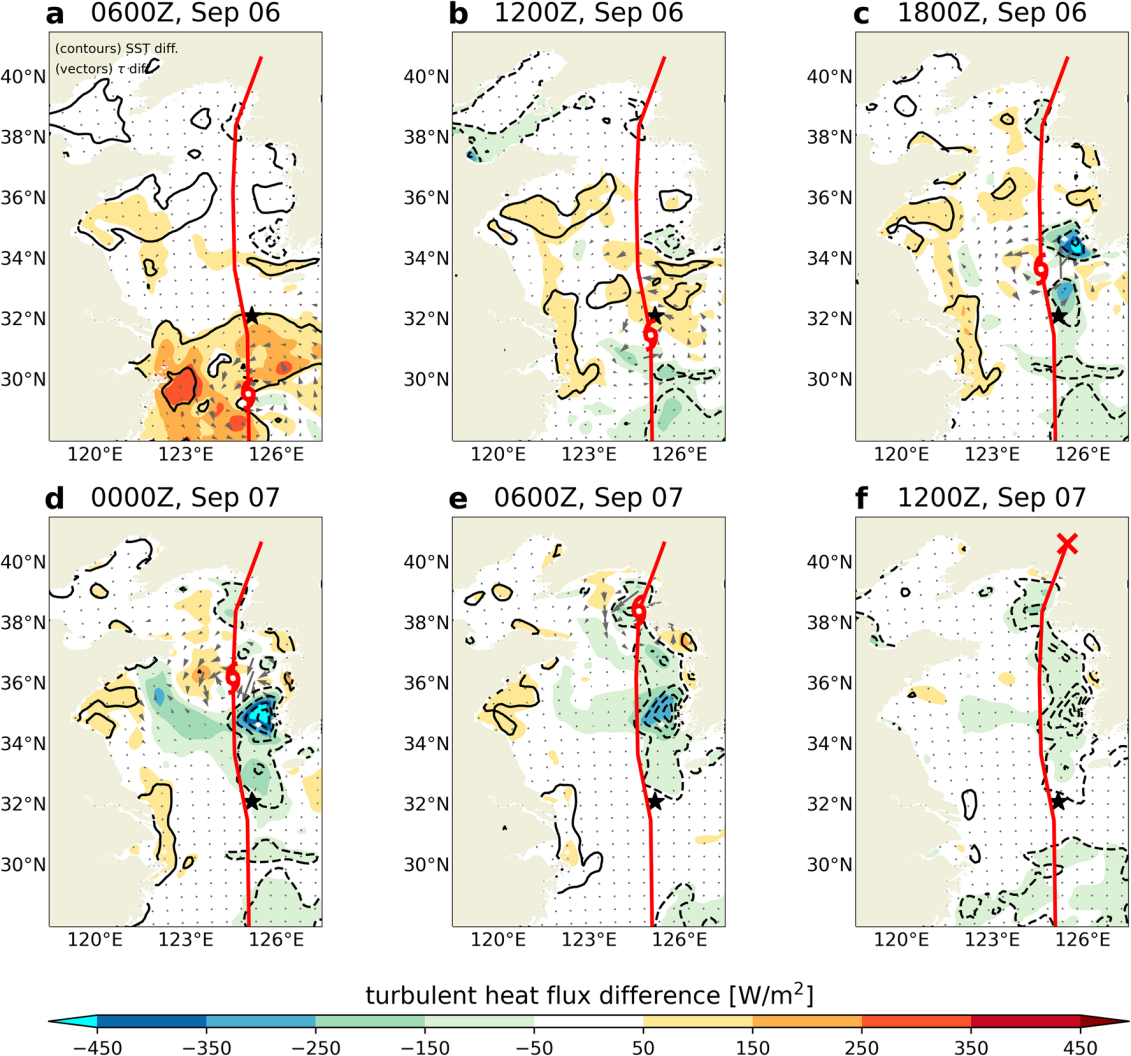
Horizontal distribution of surface turbulent heat flux differences (shaded) between CPL and NOCPL for the analysis domain every 6 h. The black contours denote sea surface temperature (SST) differences from − 6 to 6 by 2 °C. Grey arrows denote difference in wind stress (N/m^2^). Red lines and typhoon symbols indicate TC tracks and TC center location for the CPL simulation, respectively. The black star indicates the location of the Ieodo ORS.

Notably, the downward turbulent heat flux (i.e., negative heat flux) increased owing to SST cooling in the CPL simulation, wherein the sum of sensible and latent heat flux was defined as shown in Eq. (2) based on the Monin–Obukhov similarity theory^40^ in the “Methods” section. In Fig. 3, the spatial pattern of the downward turbulent heat flux was consistent with that of the SST cooling and OML deepening over the Yellow Sea because it was a function of the air-sea moisture difference (*Δq* in Eq. (2), respectively). As seen in the comparison between NOCPL and CPL (Fig. 4), the differences in wind stress mainly occurred on both sides of the TC center along its trajectory over the YSBCW. These results suggested that SST cooling in the coupled simulation affected the wind-stress differences, consistent with previous findings^43–45^. This feedback could be attributed to the differences in the turbulent heat flux. Thus, the air–sea coupling could be attributed not only to the changes in wind stress due to sea surface cooling but also to vertical structural variations in the TC center amplified by the changes in surface turbulent heat flux.

### Atmospheric response in the coupled model

**Figure 5:**
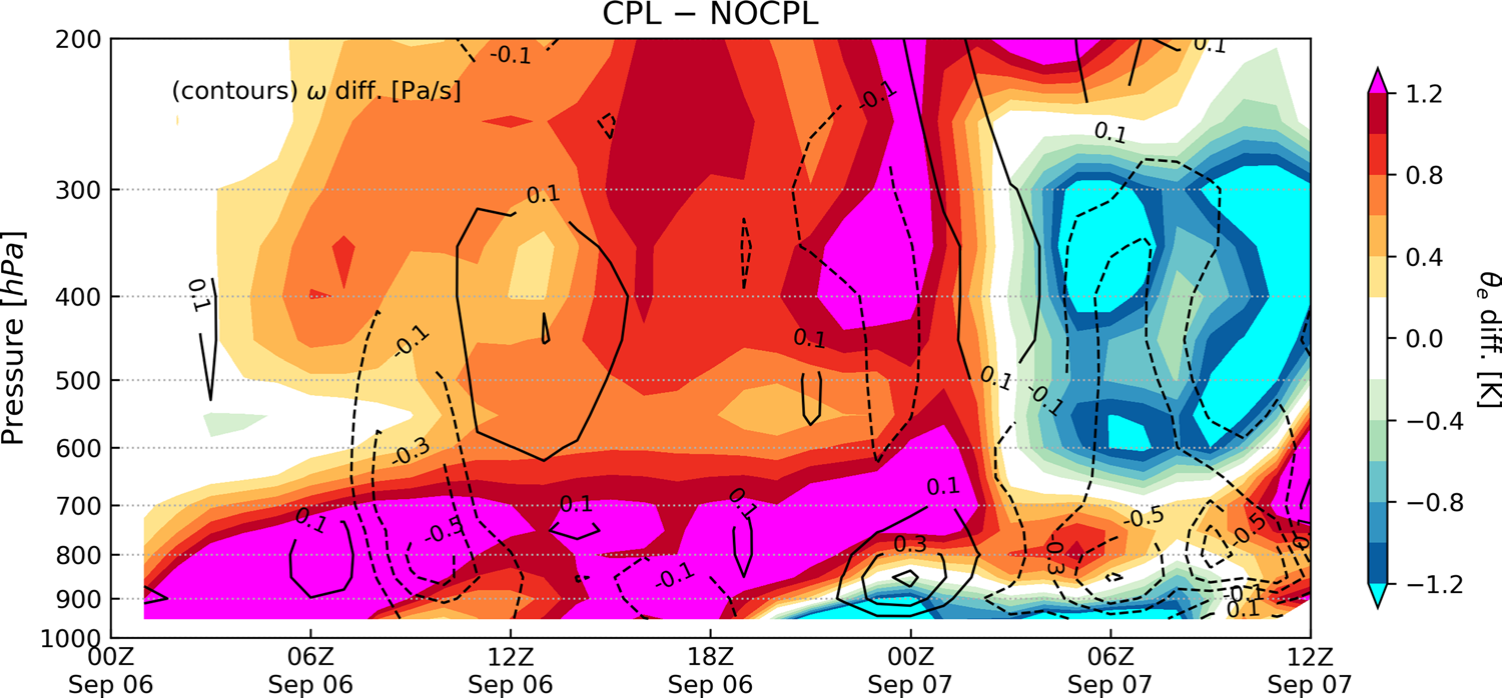
Differences of circular area-averaged equivalent potential temperature *θ*_*e*_ (K; shaded) and the vertical velocity (omega, Pa/s; contours) along the storm trajectory for CPL minus NOCPL. Radius of the area is 50 km from the TC center position in each simulation. Supplementary Fig. S4 includes the vertical velocity from both simulations.

To better understand the vertical structural variations in the TC center, we presented a time series of the theta-e and vertical velocity ${\upomega }$ along the TC passage for the difference between the uncoupled and coupled experiments (Fig. 5). The vertical velocity differences were represented by weakening (negative values) and strengthening (positive values) in the CPL compared to those in the NOCPL. Supplementary Fig. S4 shows the vertical velocity from the simulations. In the CPL simulation, the surface turbulent heat flux has an impact on the thermal structure up to 200 hPa of the region within a radius of 50 km from the TC center. The CPL simulation made the intense warm-core structure of the typhoon at the low-level atmospheric layer up to 700 hPa over the East China Sea until 1800 UTC on September 6. Following that, the SST cooling over the YSBCW was accompanied by an intense downward turbulent heat flux difference of up to approximately 561 W/m^2^ (see Fig. 4d). This change was associated with the potential temperature difference $\Delta \theta = \theta_{s} - \theta_{a}$ and specific humidity difference $\Delta q = q_{s} - q_{a}$ in Eq. (2). It affected the cooling to approximately 1 K up to the low-level atmospheric layer of 900 hPa after 1800 UTC on September 6. As the TC passed the YSBCW at 0000 UTC on September 7, the upward movement in the TC center was intensified, sending warmer and moister air than in the NOCPL simulation (see Figs. 5 and S4). The CPL simulation also showed abruptly colder air at an upper-level layer of 600–300 hPa than that in the NOCPL simulation. The cold and dry air around the TC central core abruptly spread out near the upper-level atmospheric layer of 300 hPa until 06 UTC on September 7, before landfall. Eventually, the CPL simulation supported that the upper-level atmospheric layer of 600–300 hPa resulted in cooler air with intensified downward vertical velocity than in the NOCPL simulation until the transformation of tropical to an extratropical cyclone (see Figs. 5 and S4).

**Figure 6:**
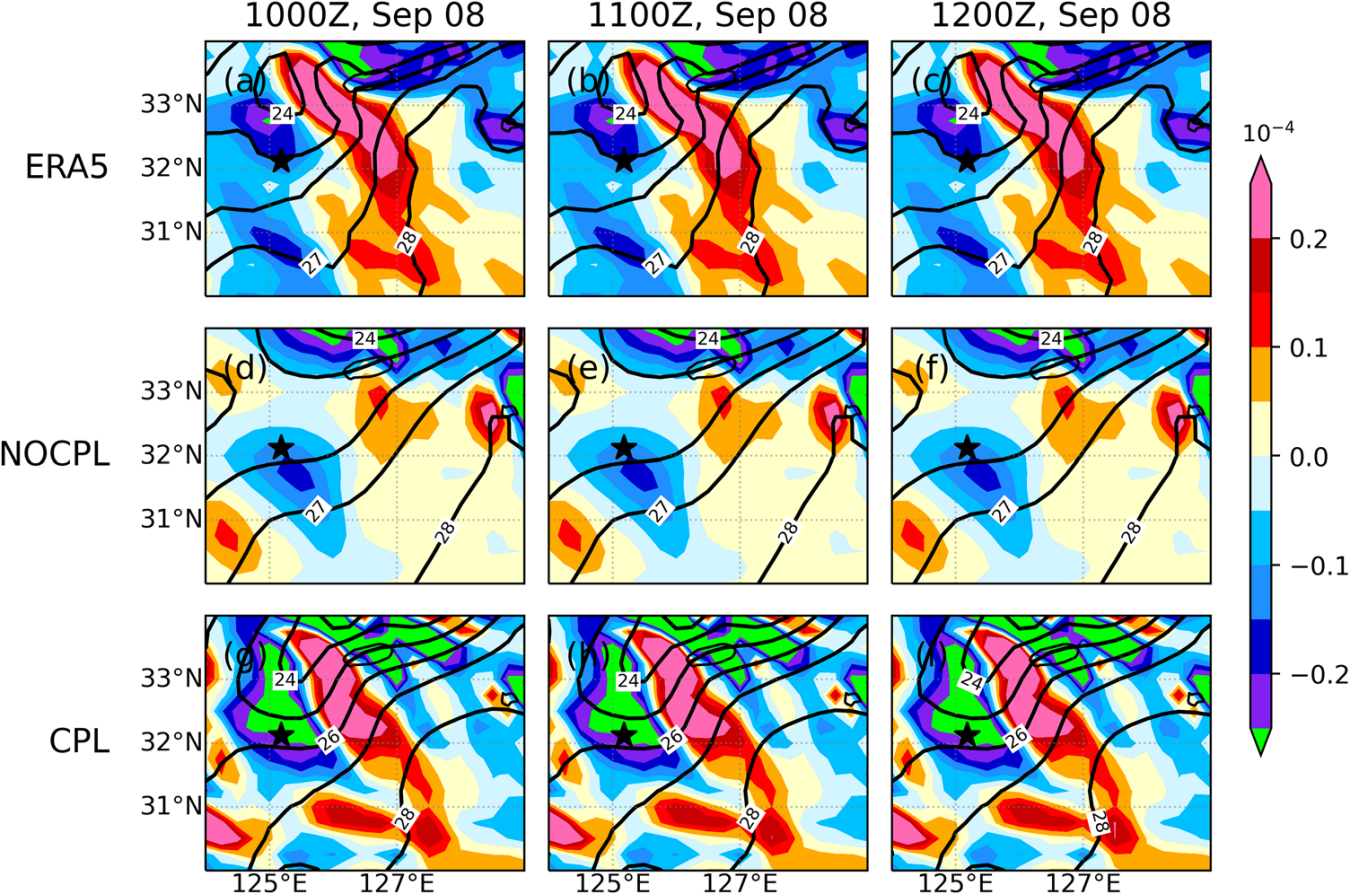
Horizontal distribution of sea surface temperature (°C; contour) and its gradient (10^–4^ K/m; shaded) for (top) ERA5, (middle) NOCPL, and (bottom) CPL every 1 h. The uncoupled and coupled results are regridded to 0.25° resolution of the ERA5 grid. The black star indicates the location of the Ieodo ORS.

**Figure 7:**
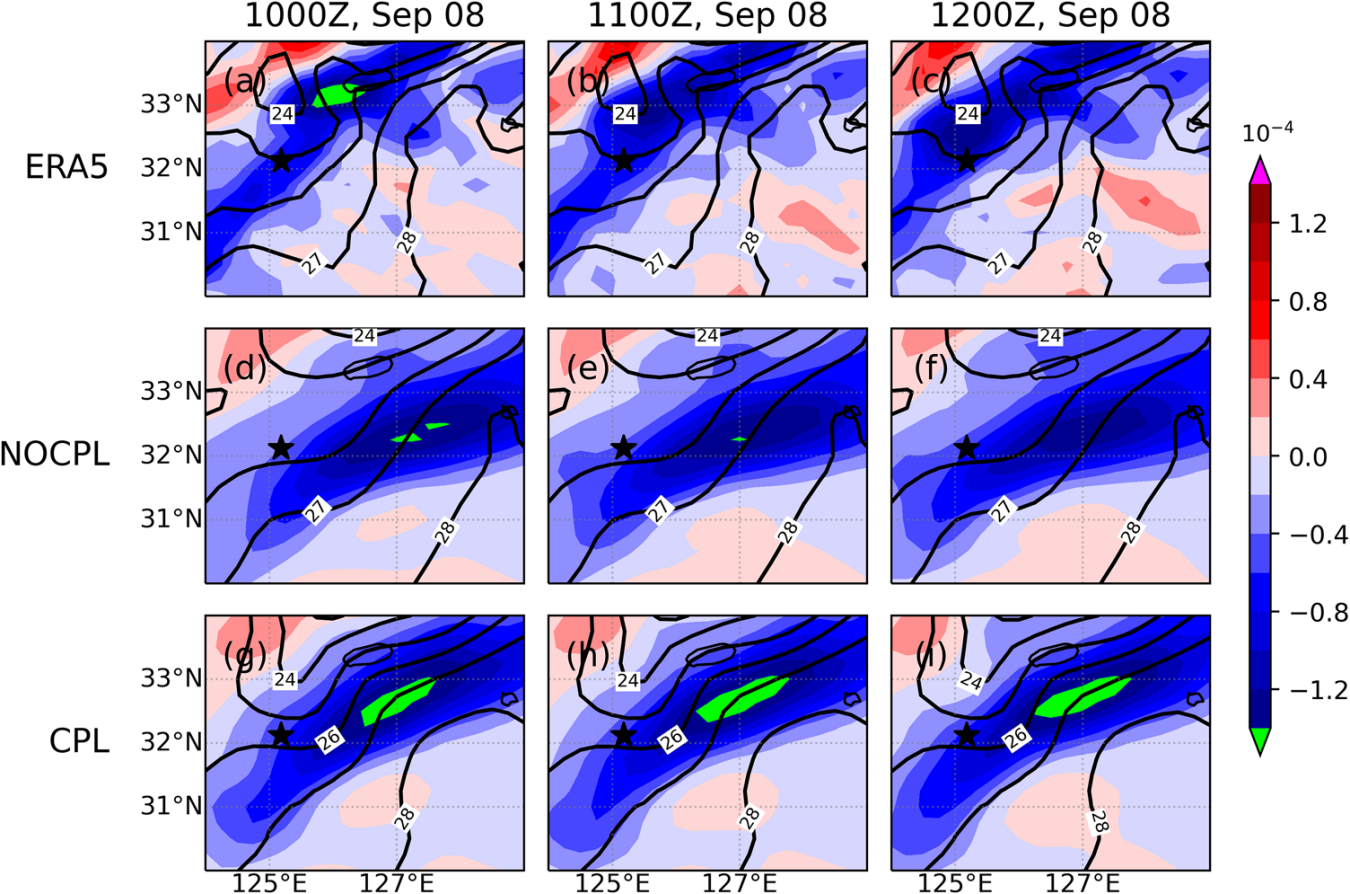
Same as Fig. 6 but for meridional gradient of 1000-hPa equivalent potential temperature (10^–4^ K/m; shaded) and sea surface temperature (°C; contour).

**Figure 8:**
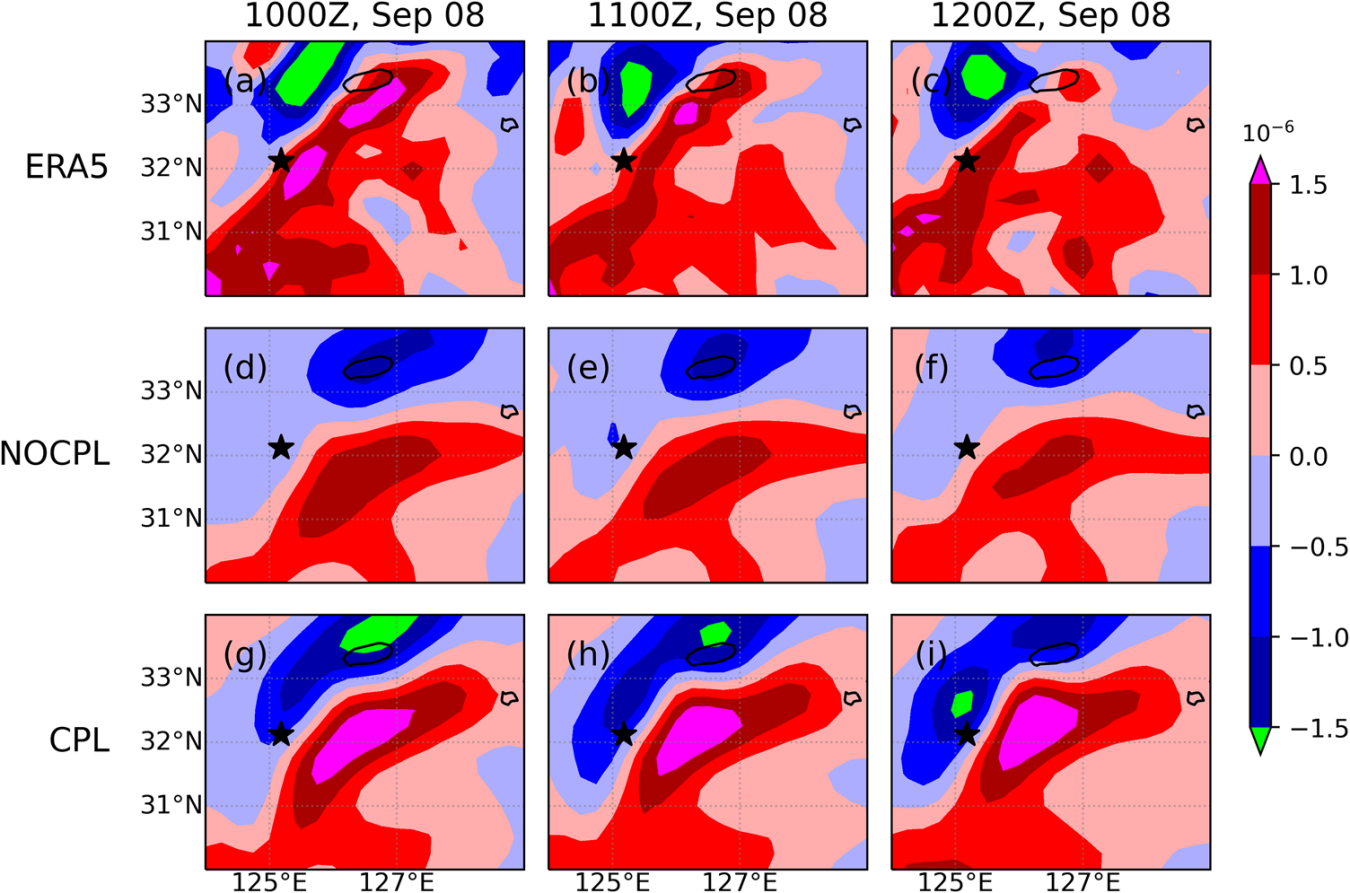
Same as Fig. 6 but for moisture convergence (10^–6^/s; shaded). The shaded regions indicate moisture convergence (positive) and moisture divergence (negative), respectively.

As shown in Fig. 5, the TC led to landfall at approximately 0900–1000 UTC on September 7 and turned into an extratropical cyclone at 1200 UTC on September 7. After the landfall, a new extratropical cyclone was observed through the East China Sea on September 8. It was accompanied by heavy precipitation up to approximately 100 mm for 12 h ahead of the warm front. In the present study, these simulation results are compared with both the Korea Meteorological Administration (KMA)'s weather radar and the ERA5 reanalysis. In the CPL simulation, SST in the YSBCW basin is decreased due to the passage of Typhoon Lingling. In addition, the CPL simulation clearly shows the relative position of the high SST gradient, which is consistent with the enhanced meridional gradient of theta-e (Figs. 6 and 7). These distributions in the CPL are more consistent with those in the ERA5. With the absence of air-sea interaction in NOCPL simulation, the baroclinic region with a weaker meridional gradient of theta-e is formed and the degree of the temperature gradient is much weaker than that of CPL simulation (Fig. 7). Moreover, Fig. S5 shows the horizontal distribution of surface turbulent heat flux for the simulation results and ERA5 reanalysis. On September 8th, the horizontal distribution of the surface turbulent heat flux was related to TC-induced SST cooling around the Ieodo ORS station. In the CPL, the surface turbulent heat flux (positive upward) was weaker than that in the NOCPL and was more consistent with that based on the ERA5 (see Fig. S5). These results indicate that the TC-induced SST cooling can support enhancement of the low-level tropospheric theta-e gradient through changes in surface turbulent heat fluxes on the ocean surface. Moreover, in the CPL simulation, moisture convergence also dominates in the region with a steep temperature gradient, which can increase rainfall amounts across the frontal zone (Fig. 8).

**Figure 9:**
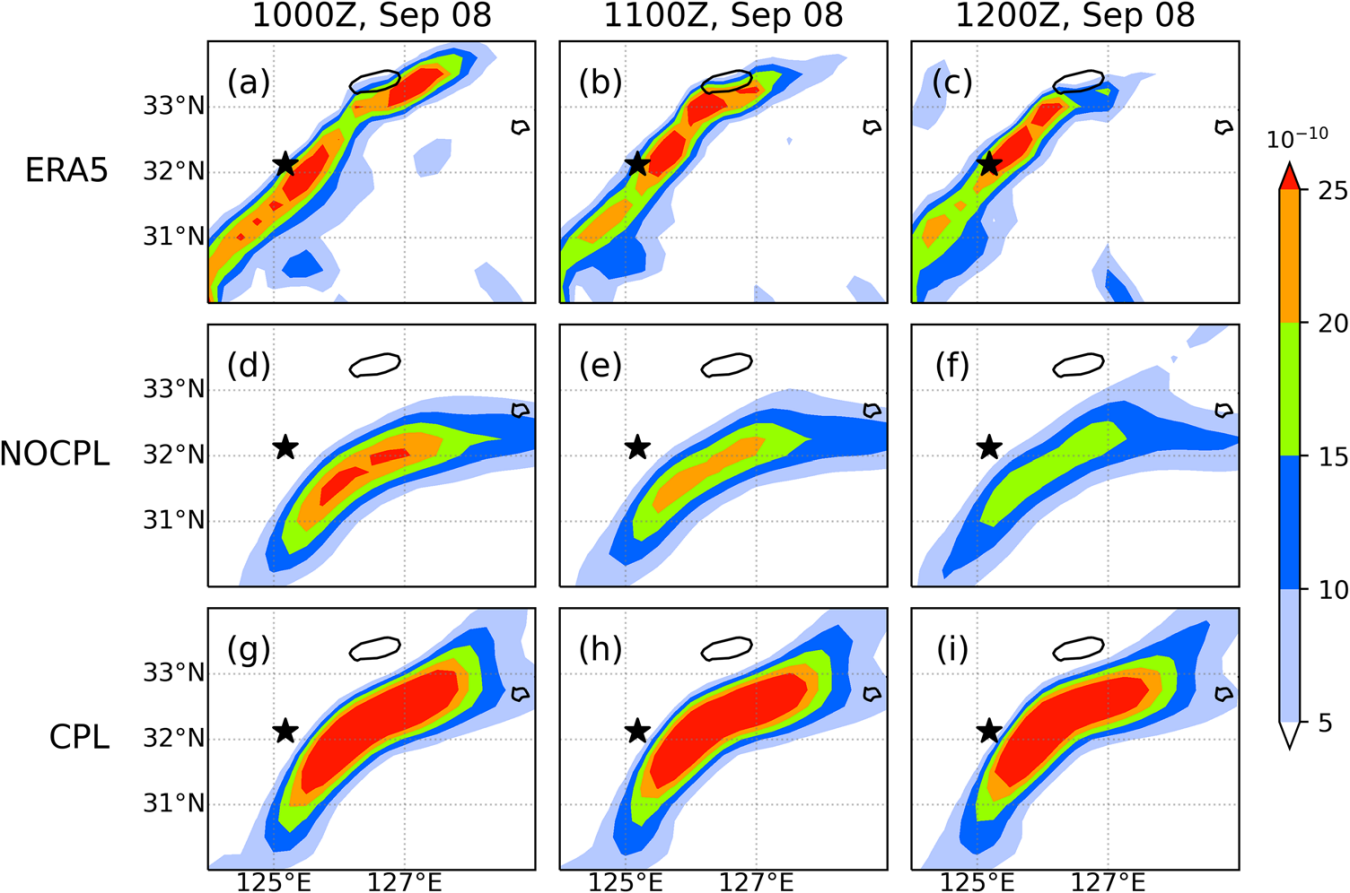
Same as Fig. 6 but for 1000-hPa frontogenesis (10^–10^ K/m s; shaded). All frontogenesis quantities are calculated by Eq. (1).

**Figure 10:**
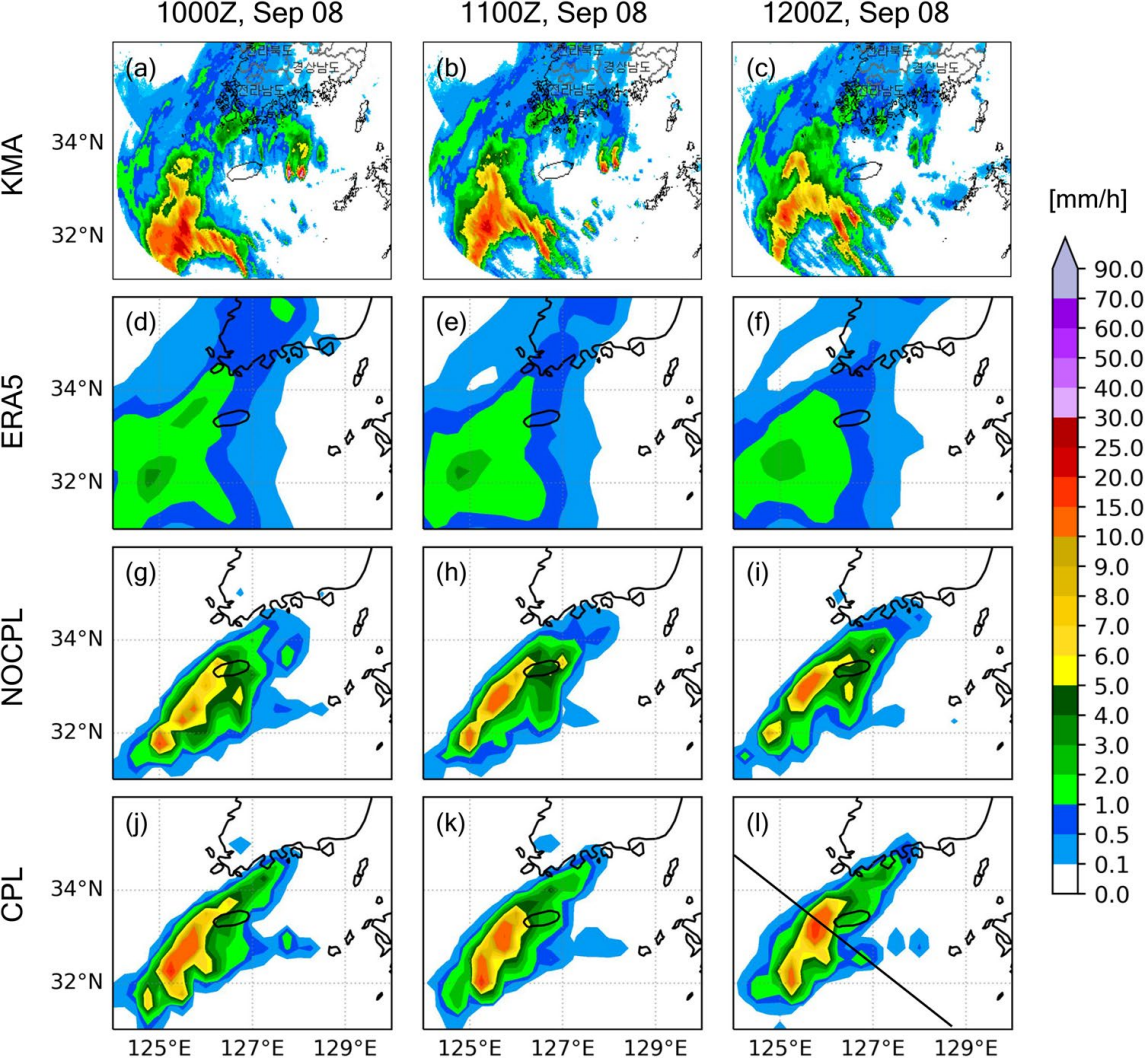
Horizontal distribution of 1 h cumulative precipitation (mm/h; shaded) for (**a**–**c**) Korea Meteorological Administration (KMA) weather radar, (**d**–**f**) ERA5, (**g**–**i**) NOCPL, and (**j**–**l**) CPL every 1 h. The solid black line on the lower-right panel (**l**) is the reference line for the cross-section of Fig. 11.

Furthermore, once the convergence zone is formed in this way, its effect can be amplified by surface heat fluxes on the ocean surface. Specifically, we analyzed the temporal evolution of 1-h cumulative precipitation and surface frontogenesis. Here, the frontogenesis was calculated as the 2D kinematic frontogenesis of a potential temperature field based on the formula outlined by Bluestein^46^. The formula is as follows:

1 $$F = \frac{1}{2}\left| {\nabla \theta } \right|\left[ {D\cos \left( {2\beta } \right) - \delta } \right],$$

where $F$ is the 2D kinematic frontogenesis, $\theta$ is the potential temperature, $D$ is the total deformation, $\beta$ is the angle between the axis dilatation and the isentropes, and $\delta$ is the divergence. Note that the sharp meridional gradient of theta-e supports the intensified frontogenesis simulated by CPL (Figs. 7 and 9). Differences in the location and strength of surface frontogenesis between NOCPL and CPL are identified (Fig. 9). From the comparison with ERA5, frontogenesis in CPL expanding from southwest to northeast south of Jeju Island is much similar to those in ERA5 compared to NOCPL (Fig. 9). Note that temperature gradient changes are related to the potential for developing not only the frontogenesis but also the band-shaped precipitation. Unfortunately, ERA5 does not capture the precipitation amounts due to its coarser grid (Fig. 10). Thus, a real radar image in KMA is used for the comparison. Whereas the NOCPL exhibited a much broader precipitation area than the Korea Strait, the CPL produces a sharper band-shaped precipitation zone around the location of frontogenesis. With the presence of the air-sea interaction in CPL, the temporal evolution of frontogenesis results in a narrow-banded precipitation zone being more similar to the real radar observation around the location of frontogenesis (Figs. 9 and 10). All these pieces of evidence support ocean coupling have a positive effect on the intensified frontogenesis close to the observed feature.

**Figure 11:**
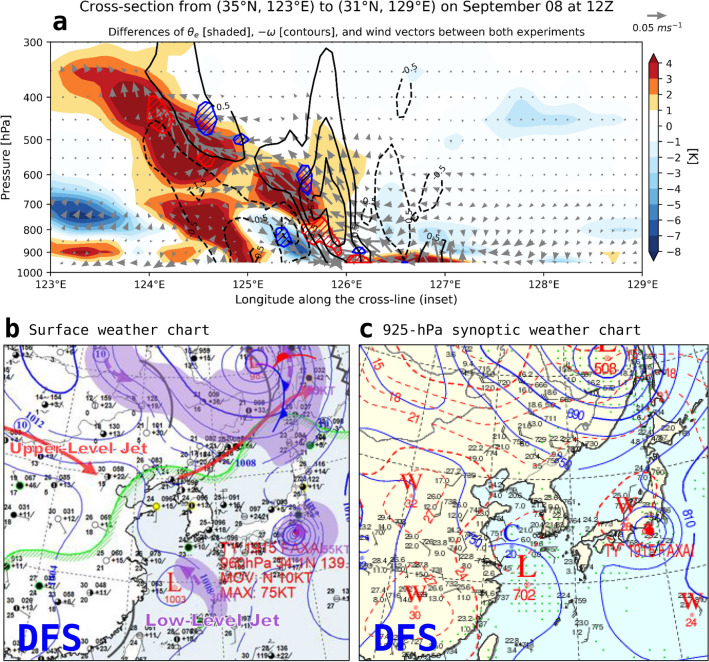
(**a**) Interpolated cross-section along the reference line on the lower-right panel of Fig. 10, (**b**) surface weather chart, and (**c**) 925 hPa synoptic weather chart on 8 Sep 2019, 12Z. The cross-section shows differences in equivalent potential temperature (shaded), vertical velocity (contours; intervals every 1 Pa/s), and moisture flux vectors. Positive shading denotes warmer air in the CPL simulation than that of the NOCPL simulation. The two weather charts in the lower panel were officially generated from the Digital Forecast System (DFS) in Korea Meteorological Administration.

Contrasting characteristics of frontal structure between CPL and NOCPL were examined in detail along a cross-line from (35° N, 123° E) to (31° N, 129° E) on September 8 at 1200 UTC (Fig. 11a). Across the front, the strengthened northwestward moist advection and ascending motion occurred through the mid-level troposphere up to 500 hPa at 125–126° E longitude. As the warm front passed over the colder air masses at the low-level troposphere up to 850 hPa, this condition allowed warmer air to rise and spread more than the NOCPL. In addition, the synoptic weather chart of the KMA confirmed the low-level tropospheric inflow of warm and moist air via the low-level jet stream toward the cold air over the Yellow Sea (Fig. 11b, c). Similarly, areas rich in moisture (i.e., positive theta-e) help to destabilize the atmosphere, which supports the generation of strong updrafts. The change in vertical motion can lead to a steeper frontal slope with an increased thermal gradient in the CPL. The steeper slope implies a greater rise and better potential for narrow banded precipitation. Thus, the surface turbulent heat flux is an essential mediator of heat and moisture, which plays a role in developing the banded precipitation over the frontal zone as well as lower-level atmospheric cooling.

**Figure 12:**
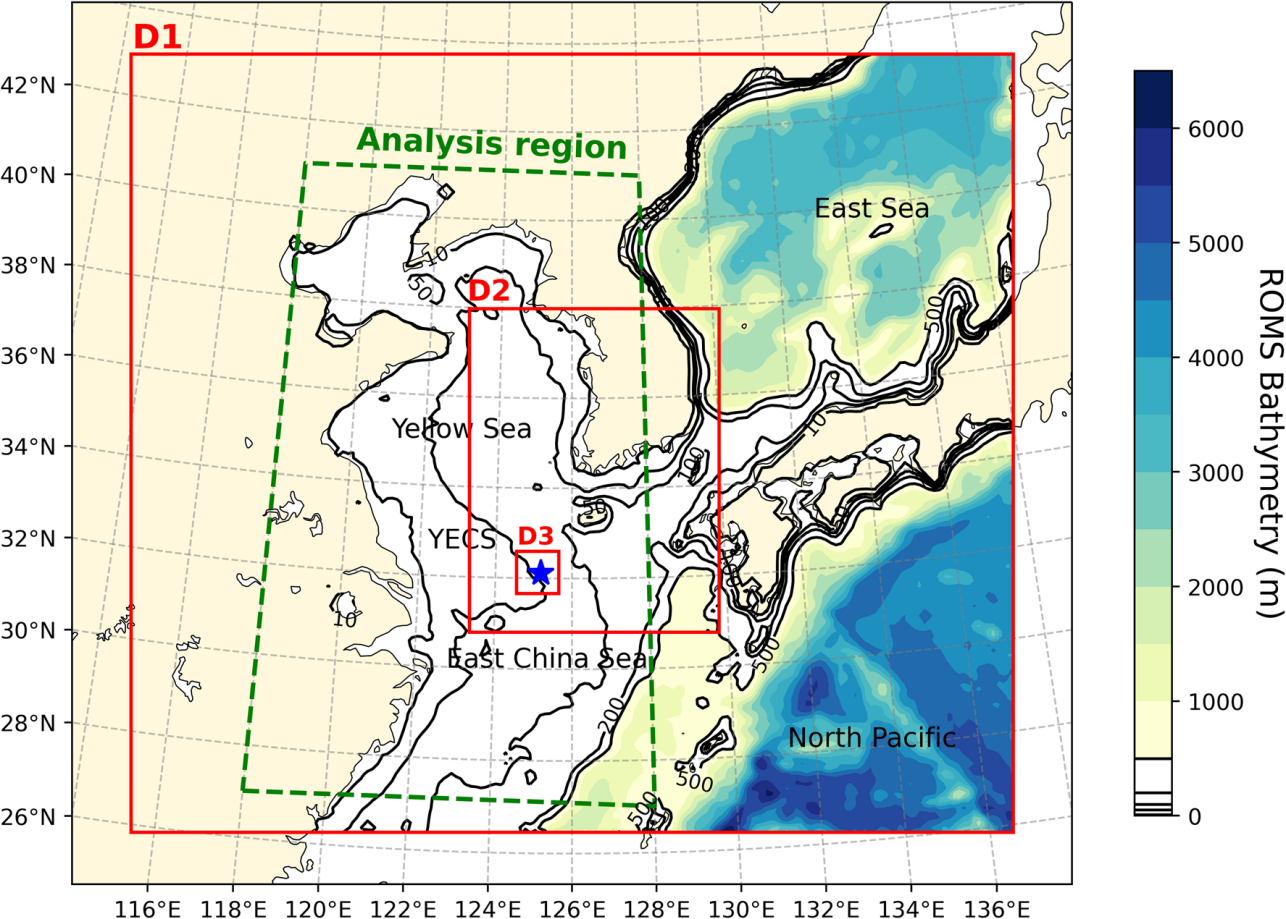
Computational model domains for WRF and ROMS. ROMS shares a mother domain of the WRF. Bathymetry under 500 m marked with contours of 50 m. Analysis region is shown by green dashed lines. The blue star indicates the location of the Ieodo ORS.

## Discussion and conclusions

In this study, we first examined how TC triggers interesting air-sea interaction phenomena over the Yellow and the East China Seas where particularly cold water exists below the mixed layer. The air-sea interaction eventually weakens TC itself in the cold basin through the downward turbulent heat flux^31,32^. Indeed, the Ieodo ORS captured the SST cooling of 4.5 °C and changes in the surface turbulent heat flux after the passage of Typhoon Lingling near the Ieodo ORS. We discussed the role of air-sea interaction in the ocean–atmosphere coupled model and how this was linked to the intensity, trajectory, and vertical thermal structure of Typhoon Lingling. Through uncoupled and coupled simulations, we confirmed: (1) the lagged oceanic response of SST cooling of 1–6 °C in the dangerous semicircle regions due to the TC-induced atmospheric forcing of wind stress and (2) changes in the vertical thermal structure of the typhoon caused by changes in surface turbulent heat fluxes.

Secondly, we highlighted the latter of our findings, which is associated with the low-level atmospheric cooling and increases in frontogenesis after the passage of TC landfall in the coupled simulation. As the typhoon passed through the YSBCW, the low-level atmospheric layer up to 900 hPa became relatively cold and dry air and greater the low-level horizontal temperature gradient. After the landfall, a new cyclone with a warm front passed through the YSBCW. In the coupled simulation, the low-level temperature gradient and moisture convergence contributed to steepening the frontal slope and causing narrow banded precipitation. Moreover, the comparison with reanalysis and observed dataset (i.e., ERA5 and weather radar image) supports the physical linkage between SST cooling and surface frontogenesis by utilizing not only the frontogenesis and precipitation but also the meridional theta-e gradient and moisture convergence zone. This analysis reveals that the coupling process indeed helps intensify the frontogenesis after the passage of Typhoon Lingling in this case study.

This research, however, is subject to several limitations. First of all, we inevitably rejected many flux observations through strict quality control to reduce anomalous information under extreme conditions. After the typhoon passage, there were large differences between observation and simulations, although a few observations were obtained as high-quality as possible. Nevertheless, it is worth noting that the differences between surface heat fluxes retrieved from observation and simulations are larger than those between the coupled and uncoupled simulations, which is not straightforward. We believe that the SST cooling in the coupled simulation was not as strong as the observation during the typhoon passage, which caused downward fluxes. Although limited, the air–sea coupling was advantageous in confirming the existence of the downward flux. Therefore, this could be a future direction for a fully coupled atmosphere–ocean model development. Second, we use a Charnock constant to calculate the drag coefficient through the friction velocity-dependent surface roughness by using a bulk formula. According to the existing field and laboratory observations^47,48^, the drag coefficient does not increase linearly with increasing wind speed under high wind conditions. That is, the wave state such as wave age (i.e., a fraction of wave phase speed and friction velocity) has an important influence on the air-sea momentum flux (i.e., wind stress)^49,50^. Another important issue is the absence of the sea spray-mediated heat flux in this study. Mainly, the air–sea coupling significantly weakens the typhoon intensity via negative SST feedback^14,51^. On the other hand, Liu et al.^27^ found that the atmosphere-wave coupling intensifies typhoon systems. This can balance the wave-related positive feedback and the negative SST feedback on typhoon intensity.

Finally, from the simulation results, we hope to enhance our understanding of atmospheric frontogenesis by air–sea coupling processes in the future development of a coupled atmosphere–ocean modeling system. However, this study deals with one typhoon, which is too limited to support a more general conclusion. Further modeling studies are needed to investigate different typhoons (e.g., slow-moving TCs such as Typhoon Soulik in 2018^18^) using a fully coupled atmosphere-wave-ocean model that includes the effect of wave and sea spray.

## Methods

### Model configuration

The COAWST modeling system was employed for this study. This coupled model consists of only the WRF and the ROMS without wave and sediment transport components in the present study. The model coupling toolkit is also utilized to interact with the WRF and ROMS in the coupled model as a coupler for every coupling time step. The coupler allows the exchange of several physical parameters among the component models. The atmospheric model transfers various atmospheric information (e.g., surface wind stress, heat flux, radiation, 10 m wind velocity, and precipitation) to the oceanic model, and the updated SST in the oceanic model is then sent back to the atmospheric model.

All simulations are carried out for the typhoon period from 0000 UTC September 6 to 12 UTC September 8. Its domain is shown with solid red lines, including the bathymetry in shades, extending from 26° to 42° N, 116° to 136° E. The analysis domain (shown in Fig. 12) covers the passage of Typhoon Lingling over the Yellow Sea and the East China Sea. The selected domain is suitable for resolving the air-sea interaction processes caused by typhoons in the present study. The WRF model selected two-way nested domains with an outer domain of 12 km and two inner domains of 3 km and 1 km. In the vertical direction, 34 sigma levels were used to discretize all domains. The WRF was initialized on September 6 at 00 UTC with the National Centre for Environmental Prediction (NCEP) Final Analysis (FNL) data using the TC initialization method^52^. We used the Monin–Obukhov similarity scheme for surface layer parameterization^40,53^ with the Yonsei University (YSU) planetary boundary layer (PBL) scheme^54^. The physics options and model configurations used in the WRF simulation are listed in Table 1.

**Table 1:** WRF configurations used in this study.

Domain	1st domain (outer)	2nd domain (inner)	3rd domain (inner)
Resolution	12 km	4 km	1 km
Horizontal grid	181 × 160	154 × 199	105 × 105
Vertical grid	34 sigma levels up to 50 hPa		
Initialization	NCEP^a^ FNL (Final) at 00 UTC September 6, 2019		
PBL scheme	Yonsei University (YSU)^54^		
Surface layer scheme	Revised MM5^b^ Monin-Obukhov^39^		
Microphysics	WRF Single-Moment 6-class (WSM6)^58^		
Cloud scheme	Kain Fritsch (KF)^59^	None	
Radiation scheme	MM5^b,^^60^ for shortwave radiation and RRTM^c,^^61^ for longwave radiation		

^a^National Centers for Environmental Prediction.

^b^Fifth-Generation Penn State/NCAR Mesoscale Model.

^c^Rapid Radiative Transfer Model.

In addition, the ROMS model was simulated with a spatial resolution of 12 km and 30 vertical sigma levels. The single domain of the model was the same as that of the outer domain of the WRF. The model's initial condition at 0000 UTC on September 6 was derived using HYCOM global analysis (https://www.hycom.org/data/glby0pt08/expt-93pt0). We mainly used generic length scale (GLS) options in the ROMS configuration with third-order horizontal advection and fourth-order centered vertical advection. For vertical mixing parameterization, the GLS vertical mixing scheme in Umlauf and Burchard^55^ was also used. One arc-min gridded global relief (ETOPO1) data from the National Oceanic and Atmospheric Administration (NOAA)^56^ was used to provide ocean bathymetry. Here, the bathymetry was smoothed using the direct iterative technique proposed by Martinho and Batteen^57^ to reduce pressure gradient errors related to sigma coordinates.

### Experimental design

In the WRF, the surface layer schemes provide a parameterization linkage for the thermal and dynamic atmospheric phenomena between the ocean and atmosphere. Among the physics options, the revised MM5 Monin–Obukhov surface layer scheme^39^ used in this study is based on the Monin–Obukhov similarity theory^40^. In the scheme, heat fluxes at the atmosphere-surface interface are given as follows:

2a $$Q_{S} = \rho_{a} c_{p} C_{h} U\left( {\theta_{s} - \theta_{a} } \right),$$

and

2b $$Q_{L} = \rho_{a} L_{v} C_{q} U\left( {q_{s} - q_{a} } \right),$$

where *Q*_*L*_ and *Q*_*S*_ are the latent and sensible heat fluxes, respectively, ${\uprho }_{a}$ is the atmospheric surface density, ${\text{c}}_{{\text{p}}}$ is the specific heat capacity at constant pressure, ${\text{L}}_{{\text{v}}}$ is the latent heat of vaporization, ${\text{U}}$ is wind speed, ${\text{C}}_{{\text{h}}}$ and ${\text{C}}_{{\text{q}}}$ are the exchange coefficients for heat and moisture, respectively. The differences in the potential temperature and specific humidity between the ocean and atmosphere are ${{\Delta \theta }} = \theta_{s} - \theta_{a}$ and ${\Delta q} = q_{s} - q_{a}$, respectively.

As mentioned above, the wind stress under extreme wind events leads to SST cooling induced by vertical mixing of the upper ocean waters along the typhoon passage. This cooling results in a downward *Q*_*L*_, exchanging moisture between the atmosphere and the ocean. However, the surface layer scheme used in this study does not allow for motion toward the ocean surface (i.e., the surface downward *Q*_*L*_ over the ocean) as follows:

3 $$Q_{L}^{ocean} = \left\{ {\begin{array}{*{20}l} {Q_{L} ,} \hfill & {if\; Q_{L} \ge 0} \hfill \\ {0,} \hfill & {if\; Q_{L} < 0} \hfill \\ \end{array} } \right.,$$

Here, we briefly modified the source code for the downward *Q*_*L*_ in the WRF. In Eq. (3), if the *Q*_*L*_ is negative, the lines that replace the zero value are excluded. Therefore, a simple flow chart of this experimental design is illustrated in Supplementary Fig. S1. We designed a coupled experiment (CPL) to investigate the effect of air-sea interaction on atmospheric frontogenesis using the modified surface layer scheme in the WRF model. The uncoupled experiment (NOCPL) was also designed to be compared with the CPL simulation. Both the coupled and uncoupled experiments were conducted for 66 h. The predefined coupling time step was maintained at 600 s through the coupler in COAWST.

In addition, we compared the flux observations in Ieodo ORS with the model results. The observation is obtained through the eddy covariance method, which is the most widely used method to obtain sensible and latent heat fluxes. The method also includes data quality control processes (e.g., spike, physical limit, and turbulent condition check). These processes have contributed to improving the quality of Ieodo ORS. For more details, see Ha et al.^1^, which describes detailed information on Ieodo ORS.

## Supplementary Information

Supplementary Information.

## Data Availability

The Ieodo ORS data are available online at http://www.khoa.go.kr/oceangrid/khoa/koofs.do through the Korea Hydrographic and Oceanographic Agency (KHOA). We gratefully acknowledge the efforts of the KHOA members in producing valuable observation data. The Joint Typhoon Warning Center (JTWC) best-track dataset is available online at http://www.usno.navy.mil/JTWC.
