# Supplementary material for: Intense atmospheric frontogenesis by air–sea coupling processes during the passage of Typhoon Lingling captured at Ieodo Ocean Research Station
Source: Sci Rep. 2022 Sep 15;12:15513. doi: 10.1038/s41598-022-19359-2 (PMC9477836; doi:10.1038/s41598-022-19359-2)
Supplement: Supplementary file 1 — Supplementary Information. [file 41598_2022_19359_MOESM1_ESM.docx]

Supplementary Information for

**Intense atmospheric frontogenesis by air-sea coupling processes during the passage of Typhoon Lingling captured at Ieodo Ocean Research Station**

**Sinil Yang1, Il-Ju Moon2, Hyo-Jun Bae1, Baek-Min Kim1,* Do-Seong Byun3, and Hwa-Young Lee3**

1Department of Environmental Atmospheric Sciences, Pukyong National University, Busan, Republic of Korea

2Typhoon Research Center, Jeju National University, Jeju, Republic of Korea

3Ocean Research Division, Korea Hydrographic and Oceanographic Agency, Busan, Republic of Korea

**Corresponding authors**: Prof. Baek-Min Kim ([baekmin@pknu.ac.kr](mailto:baekmin@pknu.ac.kr))

**This file includes**:

Supplementary Figures S1–5.

# **Supplementary Figures**

**Figure S1**. A simple flow chart of the coupled simulations for the experimental design.


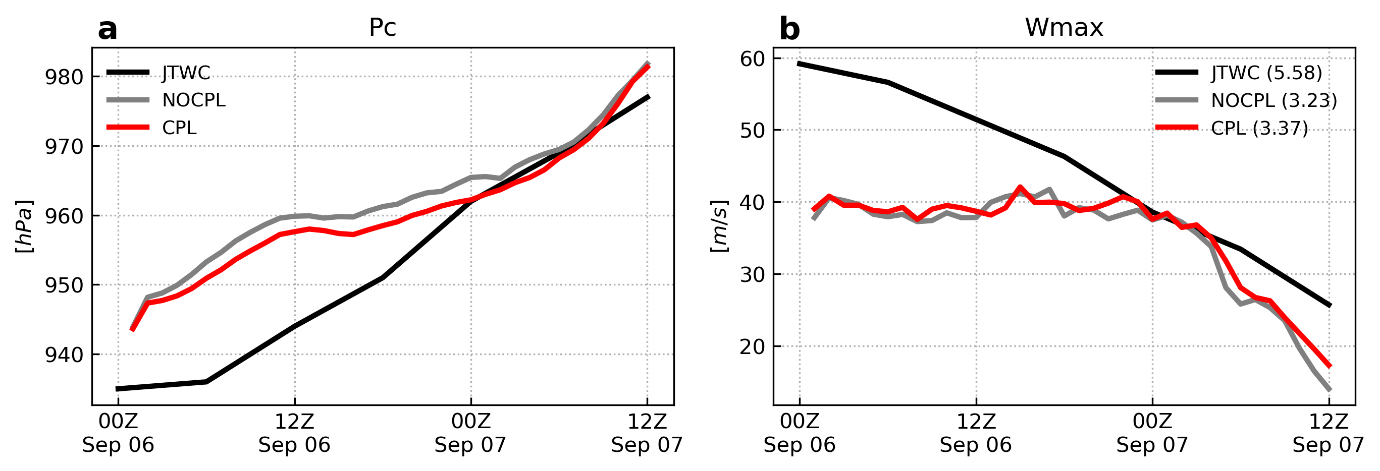


**Figure S2**. Time series of **(a)** center sea level pressure, Pc, and **(b)** maximum wind speed, Wmax, along each typhoon trajectory for (black) JTWC, (grey) NOCPL and (red) CPL (shown in Fig. 2). The value in parenthesis on the right panel is Accumulated Cyclone Energy (ACE) index for each simulation, respectively.


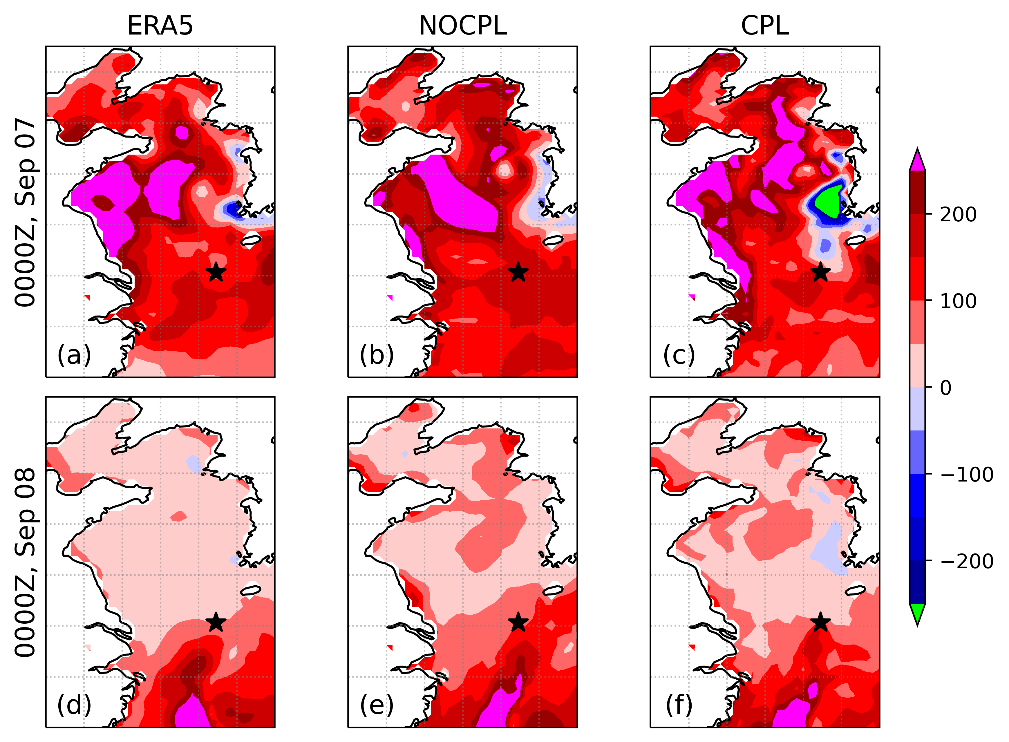


**Figure S3**. Horizontal distribution of surface turbulent heat flux (positive upward; the sum of latent and sensible heat fluxes; W m^-2^) for (left) ERA5, (middle) NOCPL, and (right) CPL. The uncoupled and coupled results are regridded to 0.25° resolution of the ERA5 grid. The black star indicates the location of the Ieodo ORS.


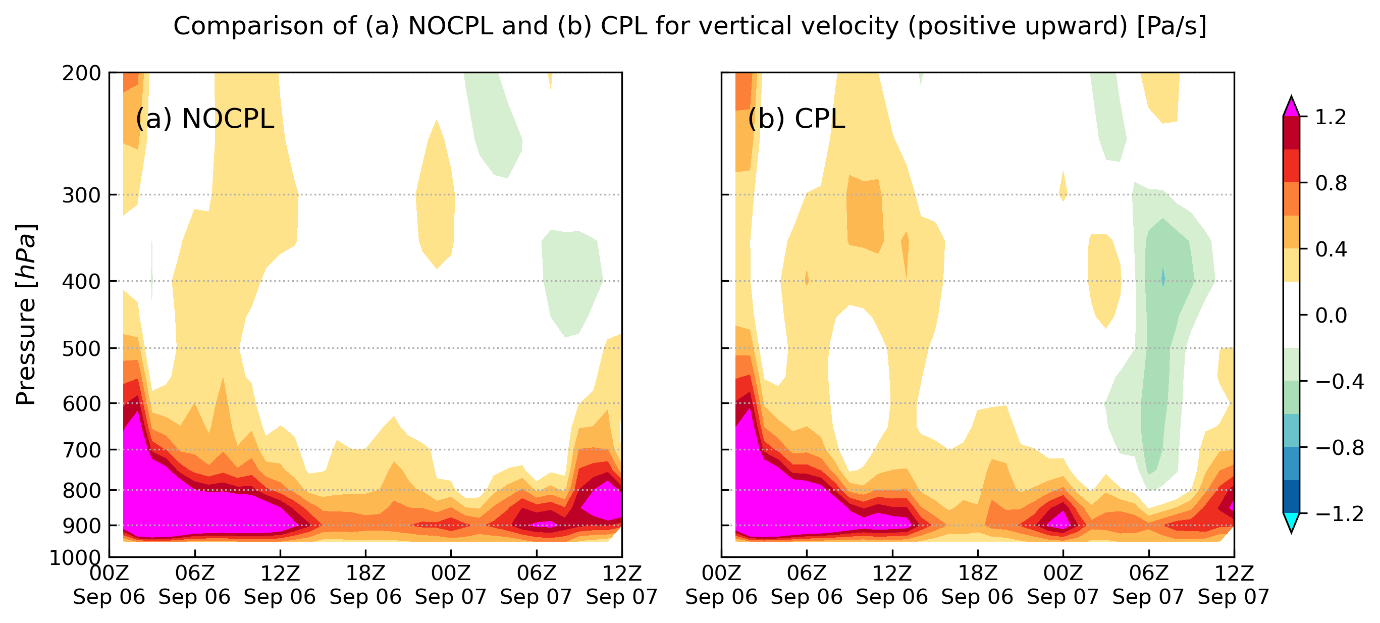


**Figure S4**. Circular area-averaged vertical velocity (Pa s^-1^; positive upward) along the storm trajectory for (a) NOCPL and (b) CPL. The radius of the area is 50 km from the TC center position in each simulation.

**
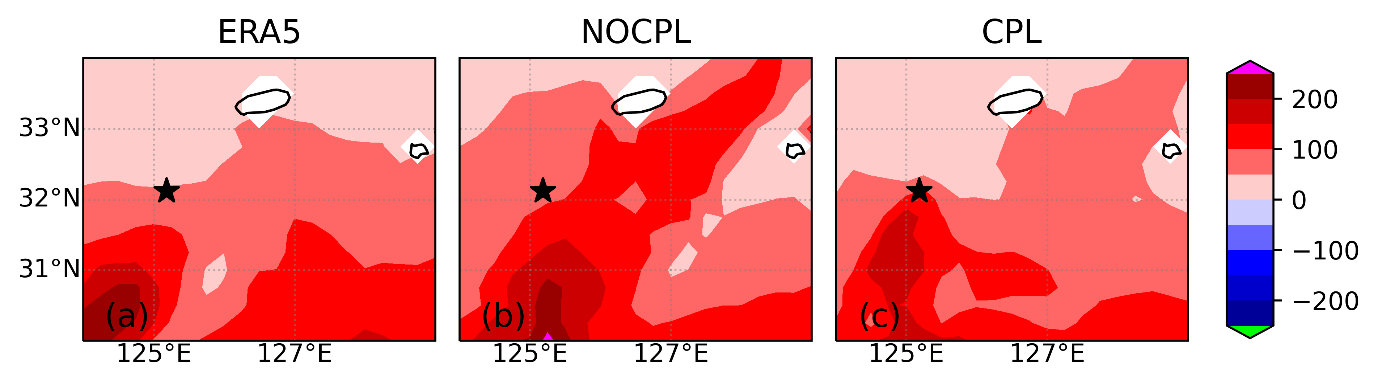
**

**Figure S5**. Horizontal distribution of surface turbulent heat flux (positive upward; the sum of latent and sensible heat fluxes; W m^-2^) for (a) ERA5, (b) NOCPL, and (c) CPL at 00 UTC on September , 2019. The uncoupled and coupled results are regridded to 0.25° resolution of the ERA5 grid. The black star indicates the location of the Ieodo ORS. This region is the same as Figure 6.
